# Melanotan-II reverses autistic features in a maternal immune activation mouse model of autism

**DOI:** 10.1371/journal.pone.0210389

**Published:** 2019-01-10

**Authors:** Elena Minakova, Jordan Lang, Jesus-Servando Medel-Matus, Georgianna G. Gould, Ashley Reynolds, Don Shin, Andrey Mazarati, Raman Sankar

**Affiliations:** 1 Department of Pediatrics, Division of Neonatology, David Geffen School of Medicine at UCLA, Los Angeles, California, United States of America; 2 Department of Internal Medicine, Huntington Memorial Hospital, Pasadena, California, United States of America; 3 Department of Neurology, David Geffen School of Medicine at UCLA, Los Angeles, California, United States of America; 4 University of Texas Health Science Center at San Antonio, Department of Cellular and Integrative Physiology, San Antonio, Texas, United States of America; 5 Children's Discovery and Innovation Institute at UCLA, Los Angeles, California, United States of America; Chiba Daigaku, JAPAN

## Abstract

Autism spectrum disorder (ASD) is a complex neurodevelopmental disorder characterized by impaired social interactions, difficulty with communication, and repetitive behavior patterns. In humans affected by ASD, there is a male pre-disposition towards the condition with a male to female ratio of 4:1. In part due to the complex etiology of ASD including genetic and environmental interplay, there are currently no available medical therapies to improve the social deficits of ASD. Studies in rodent models and humans have shown promising therapeutic effects of oxytocin in modulating social adaptation. One pharmacological approach to stimulating oxytocinergic activity is the melanocortin receptor 4 agonist Melanotan-II (MT-II). Notably the effects of oxytocin on environmental rodent autism models has not been investigated to date. We used a maternal immune activation (MIA) mouse model of autism to assess the therapeutic potential of MT-II on autism-like features in adult male mice. The male MIA mice exhibited autism-like features including impaired social behavioral metrics, diminished vocal communication, and increased repetitive behaviors. Continuous administration of MT-II to male MIA mice over a seven-day course resulted in rescue of social behavioral metrics. Normal background C57 male mice treated with MT-II showed no significant alteration in social behavioral metrics. Additionally, there was no change in anxiety-like or repetitive behaviors following MT-II treatment of normal C57 mice, though there was significant weight loss following subacute treatment. These data demonstrate MT-II as an effective agent for improving autism-like behavioral deficits in the adult male MIA mouse model of autism.

## Introduction

Autism spectrum disorder (ASD) is a complex neurodevelopmental disorder characterized by impaired social interactions, difficulty with communication, and stereotypical behavior patterns often involving repetitive activities [1]. The pathophysiology of ASD involves dysregulation of neurotransmitter expression, aberrant neuronal migration, impaired neuronal intracellular calcium signaling, and disorganization of neuronal connectivity [2]. ASD has been linked to both environmental and genetic variables. Epidemiological studies suggest maternal immune activation (MIA), especially during the second trimester increases the risk for developing ASD [3]. MIA inflammatory mouse models have been developed, which demonstrate autism-like behavior and were found to have elevation of the pro-inflammatory signal interleukin-6 (IL-6) with resultant fetal neuronal death, inhibition of neurogenesis, and impaired neuronal migration [4]. Genetic studies of ASD have identified mutations in scaffold proteins affecting synaptogenesis as well as mutations in neuronal intracellular calcium receptors resulting in abnormal axonal path-finding during brain development [5].

In part due to the complicated pathophysiology of ASD, to date no pathology directed therapies have been developed to improve the social deficits of ASD. One potential therapeutic target is the neuropeptide oxytocin. Oxytocin can alter social cognition through its ability to modulate several neurochemical systems, including serotonin, glutamate, dopamine, and GABA neurotransmitters in the hypothalamus, amygdala, and hippocampus [6,7]. Neuroimaging studies performed in human and animal models receiving inhaled oxytocin are promising for the neuropeptide’s ability to affect social adaptation by normalizing neuronal activity in key limbic regions of the brain. Oxytocin has been shown to have effects on social behavior through actions within the amygdala, dorsal raphe nucleus, hippocampus, and orbito-frontal cortex. Given the complexity of interactions within different brain regions and modulation of multiple neurochemical systems, the effects of oxytocin administration have been associated with both positive and negative social interactions including increased empathy and cooperation, but also increased anxiety and aggression depending on social context. As a result, oxytocin appears to alter social adaptation based on social cues and context [8].

Several clinical trials in humans have suggested potentially promising effects of intranasal oxytocin administration. Guastella et al. [9] showed that administration of intranasal oxytocin in a group of young males with ASD appeared to improve emotion recognition. Another clinical trial administered intranasal oxytocin to individuals with high-functioning ASD and observed an increase in visual scanning of faces along with enhanced recognition of social cues [10]. To date, several rodent models of autism have been identified as responsive to oxytocin therapy. These models include SHANK3-deficient rats [11], Cntnap2 knock-out mice [12], oxytocin receptor heterozygote mice [13], and Magel2-deficient mice [14]. Of note, Magel2-deficient mice are a key model for studying Prader-Willi-Syndrome in humans. Magel2-deficient mice have decreased oxytocin and showed significant improvement in social behaviors with oxytocin administration. The discovery led to the initiation of oxytocin clinical trials in humans afflicted with Prader-Willi Syndrome [15].

Despite these promising effects, administration of oxytocin as a therapeutic agent is challenging due its short systemic half-life ranging from 3–9 minutes given intravenously and poor blood-brain barrier permeability [16]. Also, despite some promising clinical trials in humans showing social behavioral improvement with intranasal oxytocin, there have been criticisms of the validity of the studies based on under-powering of the studies and statistical interpretations of data [17]. Furthermore, intranasal administration of oxytocin in humans and rats has yielded variability and mixed results in terms of the actual amount of oxytocin that reaches the cerebrospinal fluid (CSF) [18]. Potential novel pharmacological interventions include the use of melanocortin receptor 4 (MC4R) agonists with proven blood brain barrier permeability. MC4Rs are found on oxytocin neurons expressed within the paraventricular nucleus of the hypothalamus. Stimulation of the MC4Rs by agonists such as α-melanocyte stimulating hormone has been shown to stimulate central endogenous release of oxytocin [19]. Melanotan-II (MT-II) is an analogue of α-melanocyte stimulating hormone with a modified cyclical structure and half-life of approximately 2 hours [20]. A recent study demonstrated MT-II enhanced social cognition in prairie voles by enhancing pair bonding [20]. Also, another MC4R agonist called Ro27-3225 was shown to acutely reverse autism-like social deficits in *Cntnap2* juvenile mice [12].

We tested the effects of MT-II on social behaviors of adult MIA male mice via a seven-day intra-ventricular infusion and found significant improvement in social behavioral metrics. We also administered the MT-II infusion over a fourteen-day course to background C57BL/6J mice to assess for any side effects associated with subacute administration of MT-II, along with any alterations in social or non-social behaviors following MT-II administration. We did not find significant changes from baseline in social or non-social behaviors in the background C57BL/6J mice following MT-II administration.

Given the exquisite sensitivity to endogenous oxytocin stimulation and improvement of social deficits in the MIA mice, we also evaluated for oxytocin receptor brain expression in the male MIA mice and the typical-behaving C57BL/6J mice. We noted a significant increase of oxytocin receptor expression in the anterior cingulate cortex in the MIA mice, which is a region of the brain with a key role in socio-emotional processing.

## Materials and methods

### Animals

C57BL/6J mice (Charles River, Hollister, CA) were maintained in standard laboratory conditions. Mice were mated and the presence of a vaginal plug was indicative of embryonic day 0 (E0). Pregnant mice were housed separately for the remainder of the pregnancy. The maternal immune activation (MIA) mouse model was generated as previously described [21]. Briefly, to generate MIA mice, pregnant C57BL/6J mice received a daily 30 μg/kg intraperitoneal injection of recombinant IL-6 (R&D Systems, Minneapolis, MN) during E12.5–16.5. The dosage was selected based on a dosage response curve published by Pineda et al. [22]. The male offspring were subsequently used for experiments—referred to as MIA mice in this manuscript. Four- to six-month-old male MIA and male control C57BL/6J mice (Charles River, Hollister, CA) were used in this study. Animals were kept at 12-hour light (7:00 AM- 7:00 PM)/ dark (7:00 PM- 7:00 AM) cycles with ad libitum access to food and water. Experimental procedures were approved by the UCLA Animal Research Committee and complied with NIH and AAALAC guidelines and requirements.

### Melanotan-II treatment

#### Experimental drug and delivery system

Melanotan-II (MT-II) [Ac-Nle-cyclo[Asp-His-D-Phe-Arg-Trp-Lys]-NH_2_] (Sigma Aldrich, St. Louis, MO) stock solution was prepared by dissolving MT-II in sterile water at a concentration of 5 mg/ml. For treatment of MIA mice, MT-II was further diluted in 0.9% saline and continuously administered into the lateral ventricle for 7 days via a surgically placed cannula (Brain Infusion Kit 3) with controlled delivery by the Alzet micro-osmotic pump model 1007D (Durect, Cupertino, CA). Similarly, for subacute MT-II treatment of control C57BL/6J mice, MT-II was continuously administered for fourteen days using the Brain Infusion Kit 3 with the Alzet osmotic pump model 1002D.

In all treatments, MT-II was administered at 2.5 μg per day with dosing based on previous studies showing physiological effects at the above dosing range value [20, 23]. Based on the ranges provided in the literature, we decided to empirically use the 10 mg/kg systemic dose and converted the amount of drug delivered to a more appropriate level for intracerebroventricular administration. Since the adult mouse brain weighs ~ 0.4 grams and the average weight of our adult C57BL/6J male mice ranged from 25–30 grams, the brain weight to total body weight ratio was approximately 1:100. The 10 mg/kg systemic dosing was decreased to a dose of 0.1 mg/kg for intracerebroventricular administration. For vehicle controls, 0.9% sterile saline alone was administered.

### Surgery

Mice were anesthetized with isoflurane and placed in the mouse adapter (Stoelting, Wood Dale, IL). A subcutaneous pocket was created in the dorsum and an Alzet pump was implanted. The cannula from the Brain Infusion Kit 3 was stereotactically placed into the left lateral ventricle at the following coordinates from Bregma: posterior 0.20 mm, left 0.8 mm, ventral 2.5 mm. The cannula was fixed to the skull using the Cyanoacrylate Adhesive (Rocky Hills, CT) and the incision was closed using 4–0 sutures. After completion of experiments, mice were euthanized with pentobarbital and underwent perfusion with PBS followed by 4% paraformaldehyde. Brains were removed, underwent a series of alcohol dehydrations, and were embedded in paraffin. Eight μm coronal sections were obtained and confirmed placement of the cannula within the left lateral ventricle.

### Behavioral testing

Behavioral testing was performed between 10:00 AM and 4:00 PM in a noise insulated, low-lit room. Animal behavioral testing was video-taped and analyzed offline.

### Self-grooming test

Mice were placed into a clean beaker (10 cm height x 6.5 cm width) and habituated for 10 minutes [24]. Following habituation, grooming time was recorded over a 10-minute session. Licking of the fur and scratching with paws were counted as grooming behavior. The behavioral test was performed in a dimly lit room with observer separated by a 2-meter distance. The beaker was rinsed with water and cleansed with ammonium chloride before testing.

### Three chamber test

The three-chamber test was performed in a 60 cm x 40 cm Plexiglas box (Noldus, Leesburg, VA) divided into three connecting chambers as described by Hagen et al. [25]. The end of each compartment contained a wired cylindrical enclosure (11 cm height x 10 diameter, bar space 1 cm apart). Prior to initiation of testing, the test mouse was placed into the center of the chamber and allowed to explore all three chambers. After the 10-minute habituation period, the test mouse was placed into the center chamber. For sociability testing, an unfamiliar age-, strain-, and sex-matched animal was placed under one enclosure while a plastic bottle cap was placed in the other enclosure. The center chamber was opened and the test mouse was allowed to explore all three chambers for 10 minutes and time spent near the object or mouse was recorded. For social novelty testing, the test mouse was placed into the center chamber and a novel conspecific mouse replaced the object. The test mouse was then allowed to explore all three chambers for 10 minutes and time spent with the stranger versus familiar mouse was recorded. For all three-chamber tests, conspecifics or the object were randomly assigned to either chamber. Analysis of behavior was expressed as sociability index (first phase), which involved measuring the time (t) in seconds spent interacting with the conspecific and object and calculation by the formula: {[t_conspecific_/ (t_conspecific_ + t_object_)] X 100}– 50. Social novelty (second phase) was expressed as social novelty index and calculated as follows: {[t_new conspecific_/ (t_new conspecific_ + t_old conspecific_)] X 100}– 50. The sociability and social novelty index scores span from a score of -50, indicating complete avoidance of the conspecific during phase I and complete avoidance of the new conspecific during phase II, to a score of +50, indicating complete preference for the conspecific during phase I and complete preference for the new conspecific during phase II; a score of 0 indicates social indifference [24].

### Ultrasound vocalization testing

Baseline adult male-female social interactions were investigated using ultrasound vocalization (USV) testing. Adult male MIA mice and female control C57BL/6J mice were habituated to their home cages for a period of five days prior to USV recording. To elicit a novel situation, the male mouse and female mouse (in estrous) were placed into a clean cage with ~1.5 cm bedding thickness [26]. The cage was then placed into a noise-limiting chamber (40 cm length x 28 cm width x 30 cm height) with the ultrasonic microphone (Ultrasonic Microphone and Amplifier FRI-100-1, Florida Research Instruments, Cocoa Beach, Florida) suspended 15 cm above the cage. Frequency sensitivity was set at 10 to 180 kHz; signals below 30 kHz were excluded as noise. USVs were recorded over a five-minute session using RavenPro 1.4 Software, which was subsequently used to analyze USV incidence.

In general, there is an overall assumption that male mice produce the USVs during courtship with some studies suggesting that the female mouse does not vocalize in the presence of an anesthetized male while the converse is true if a male mouse is around an anesthetized female mouse. However, there is existing literature documenting that both males and females produce USVs during courtship rituals. One study using a microphone-array-based system to help localize the source of USVs between paired interactions confirmed males and females both vocalize during courting [27]. We did not have a microphone-array-based system to allow for specific assignment of each call, so all USVs were attributed to the male mouse.

### Exploratory behavior

The test was performed in an open-square black arena (40 cm x 40 cm x 30 cm) with the floor divided into 25 squares, as described previously [28]. The number of lines crossed over a five-minute period was indicative of exploratory behavior [29]. Crossings were counted by reviewing the video recording from an overhead camera. Prior to each testing session, the arena was cleaned thoroughly with 70% ethanol.

### Marble burying

Subject mice were habituated to a clean cage (27 cm length x 16 cm width x 12 cm height) filled with ~ 5 cm thickness of wood-chips over a 15-minute period. Afterwards, 20 clean glass marbles were placed into the cage equidistant from each other. Mice were then left with the marbles for 30 minutes, and after this period marbles were counted as buried if > 50% of the marble was covered with bedding [30].

### Oxytocin receptor autoradiography

Since injection of recombinant IL-6 occurs at a time period of fetal mouse oxytocin receptor expression development (E12.5–16.5), we performed oxytocin receptor autoradiography in the adult MIA male mice and the typical-behaving C57BL/6J male mice brains. Oxytocin receptor expression was measured in the following brain regions: prelimbic cortex, anterior olfactory nucleus, nucleus accumbens, lateral septal nucleus, bed nucleus of stria terminalis, ventral reuniens, paraventricular and ventromedial hypothalamic nuclei, hippocampal CA1 and CA2/3 regions and the central amygdaloid nucleus.

Binding of iodinated ornithine vasotocin analog ([^125^I] OVT) to oxytocin receptors was performed following methods from Insel et al. [31] and Artymyshyn et al. [32] with minor modifications for use in mouse brains. Coronal sections of 16 μm were collected from brain regions of interest (per Paxinos and Franklin, [33]) at -16°C in a cryostat (Leica, Banockburn IL) and thaw-mounted onto gelatin coated microscope slides. Sections on slides were vacuum-desiccated for 18–24 h at 4°C, then stored at -80°C until use. Brain sections were thawed for 30 min at 23°C, and then pre-incubated for 30 min in 50 mM Tris HCl buffer pH 7.4 at 23°C. Mounted sections were next incubated for 90 min in cytomailers with buffer containing 10 mM MgCl_2_, 0.1% bovine serum albumin and 100 pM [^125^I] OVT (NEX2540, PerkinElmer, Boston MA). Non-specific binding was determined by incubating representative sections on slides from the series in incubation buffer containing unlabeled oxytocin (1 μM, Ascent Scientific, Bristol UK). Sections were washed twice for 5 min each in staining dishes containing 4°C in incubation buffer, and were next dipped for 2 sec in 4°C deionized water. Slide backs were tapped on padding and dried with paper towels and then placed section side up on a benchtop slide warmer, setting 4 for 1 h or until sections were opaque and all droplets had dissolved. The sample size consisted of five brains per treatment group of mice.

Sections on slides were opposed to Biomax MR film (Carestream/Kodak) for 3 days along with tritium standards previously calibrated to brain mash incubated in ^125^I [32]. Digital images of autoradiograms were captured using a 12 bit monochrome digital camera (1394 Scion Corporation, Frederick, MD) with a 28 mm lens (f-stop = 4, Nikon, USA) mounted on a copy stand, and a precision illuminator (Northern Lights, MCID, UK) with image J software (https://imagej.nih.gov/ij/download.html).

### Experiments

The MIA mice received MT-II or vehicle infusion over seven days with the Alzet pump. Baseline three-chamber testing was performed seven days before pump implantation. Following pump implantation with vehicle or MT-II, repeat three-chamber testing was performed on day seven of the infusion.

The normal C57BL/6J mice received the infusion over an extended fourteen-day time period to assess for any changes in social behavior using the three-chamber test and also to assess for any behavioral side effects or weight loss associated with MT-II. Extension of drug duration administration to fourteen days allowed for adequate spacing between tests to perform open field anxiety testing and marble burying along with monitoring for weight loss. Since MIA mice have behavioral impairments at baseline, we did not extend their infusion for fourteen-days to assess for potential behavioral side effects of MT-II. Normal C57Bl/6J mice had baseline three-chamber testing performed seven days before pump placement, the open field anxiety test was performed six days prior to pump placement, marble burying was performed five days prior to pump placement, and weight was measured immediately following pump placement. Table 1 shows the timeline of baseline behavioral testing in normal C57BL/6J prior to implantation of mini-pump containing vehicle or MT-II. Following implantation of the pump, three-chamber testing was performed on day seven of infusion, OFA test was performed on day nine, marble burying was performed on day fourteen and weight was also measured on day fourteen of the infusion. Table 2 shows the timeline of behavioral testing in normal C57BL/6J mice during the fourteen-day infusion of vehicle or MT-II. The mice were tested in randomized orders during the experiments.

**Table 1:** Normal C57BL/6J mouse baseline experimental testing timeline.

BL 3-CT	OFA BL Testing	Marble Burying BL Testing	Weight Analysis
Performed 7 days before mini-pump implantation	Performed 6 days before mini-pump administration	Performed 5 days before mini-pump implantation	Performed immediately following pump implantation

Abbreviations: BL, baseline; 3-CT, three chamber test, OFA, open field anxiety test. The table illustrates the baseline testing in normal C57BL/6J mice with the relative timing of the tests prior to mini-pump placement containing vehicle or MT-II. Following the completion of baseline testing, the mice were implanted with the pump and weighed immediately post-operatively.

**Table 2:** Normal C57BL/6J mouse experimental testing timeline during vehicle or MT-II infusion.

Infusion with vehicle or MT-II Days 1–6	Infusion with vehicle or MT-II Day 7	Infusion with vehicle or MT-II Day 8	Infusion with vehicle or MT-II Day 9	Infusion with vehicle or MT-II Day 10–13	Infusion with vehicle or MT-II Day 14
No testing	Three chamber test performed	No testing	OFA testing	No testing	Marble burying testing and weight measurement

Abbreviations: BL, baseline; 3-CT, three chamber test, OFA, open field anxiety test. The table illustrates the timing of behavioral tests performed following surgical implantation of the mini-pump containing vehicle or MT-II. Other than the behavioral tests, the weight following sub-acute infusion of the drug was obtained on the last day of the infusion, day fourteen.

### Data analysis

Data was analyzed in a blinded fashion using Prism 6 software (GraphPad, San Diego, CA). Paired and unpaired Student T-tests were used to analyze parametric data. One-way ANOVA was used to analyze treatment interactions between MIA mice followed by the Sidak’s multiple comparisons test. Two-way ANOVA was used to analyze for treatment-interaction effects between MIA mice and normal C57BL/6J mice. Multivariate Analysis of Variance with Newman Keuls post-hoc was performed to compare oxytocin receptor expression in the brain regions of interest between untreated MIA mice and normal C57BL6/J mice groups using Statistica software (Statsoft, Tulsa OK). Statistical tests and sample numbers for each experiment are indicated in figure legends. Results are reported as the average +/- standard error of the mean, unless otherwise noted.

## Results

### Maternal immune activation mice exhibited impaired social interaction, decreased communication, and repetitive behavior

Current epidemiological evidence suggests that maternal immune activation (MIA) secondary to infection during pregnancy may be an environmental risk factor predisposing towards the development of ASD [3, 34, 35, 36, 37, 38]. Multiple autism rodent models of MIA have been described in the literature including mid-gestation maternal exposure to lipopolysaccharide (bacterial mimic), Poly I:C (viral mimic), or IL-6 [2, 35]. We chose to induce MIA via mid-gestational treatment with recombinant IL-6, a cytokine that has been shown to be a key mediator in provoking detrimental gene dysregulation in the fetal brain associated with MIA [21].

Adult male MIA mice, the offspring of C57BL/6J pregnant mice treated with IL-6 during mid-gestation, underwent behavioral testing to assess for core behavioral features associated with ASD. These features include deficits in social interaction, impaired communication, and repetitive behavior, which were evaluated by three chamber testing, ultrasound vocalization (USV) testing, and self-grooming testing, respectively. The metric scores of MIA mice were compared to age-matched control C57BL/6J male mice.

**Fig 1:**
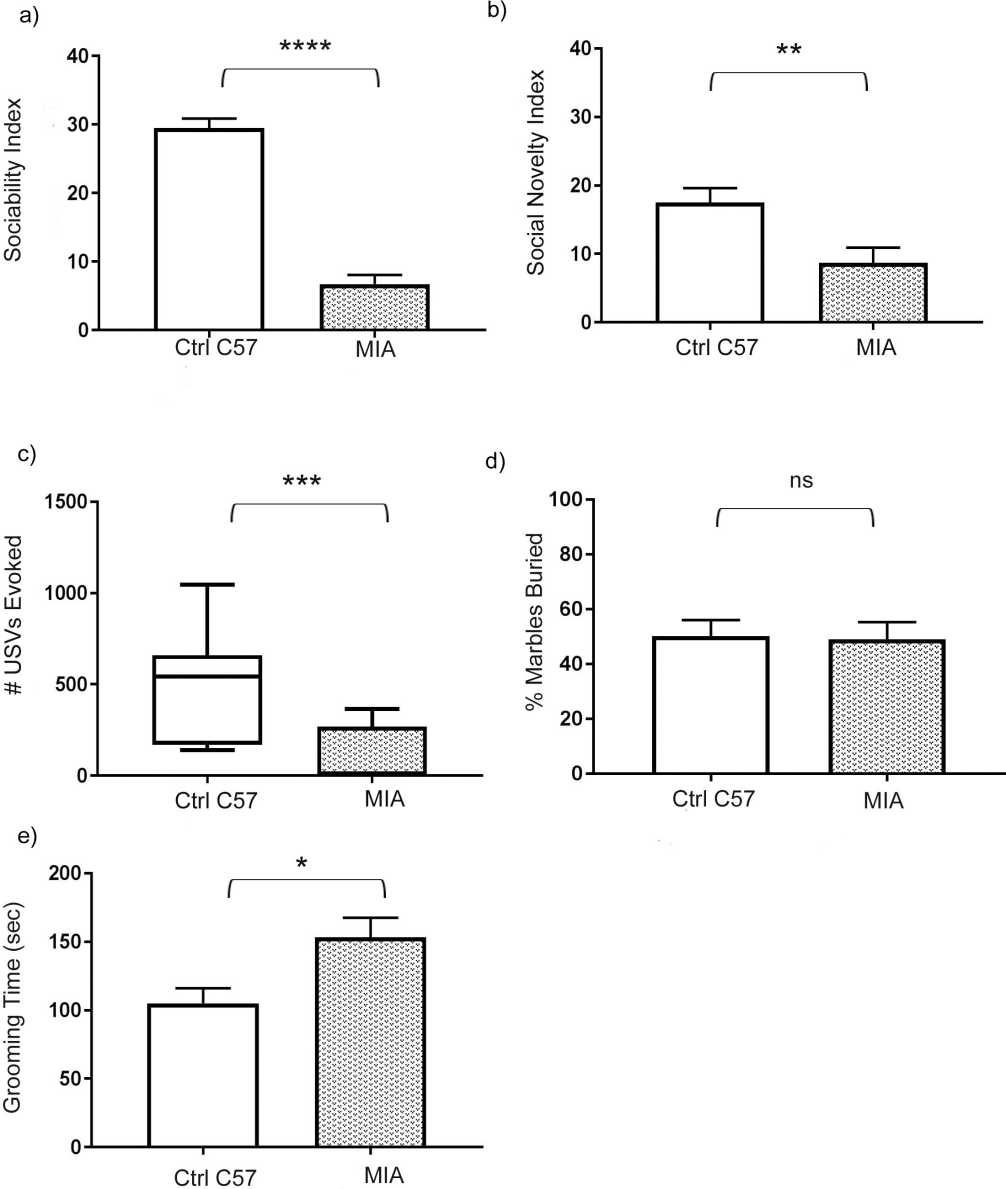
Maternal immune activation mice exhibited impaired social interaction, decreased communication, and repetitive behavior. (a) Three-chamber test assessment of sociability index, with results shown for maternal immune activation (MIA) mice and control C57BL/6J mice (control mice, n = 24; MIA mice, n = 35). Student’s unpaired *t* test, p < 0.0001. (b) Three-chamber test assessment of social novelty index, with results shown for MIA and control mice (control mice, n = 24; MIA mice, n = 34). Student’s unpaired *t* test, p = 0.0077. (c) Ultrasound vocalizations (USVs) were recorded during interactions with the male test mouse in the presence of an unfamiliar female in estrous. The number of USVs recorded in a five-minute period are shown for MIA and control mice. USVs are graphed as box-whisker plots (control mice, n = 7; MIA mice, n = 15). Mann-Whitney test, p < 0.001. (d) Marble burying assessment of MIA and control mice. The percentage of marbles buried at the end of a thirty-minute period is shown (control mice, n = 22; MIA mice, n = 24). Student’s unpaired *t* test, p = 0.88. (e) Grooming assessment of MIA mice and control mice. The time in seconds spent grooming over a ten-minute period is shown (control mice, n = 15; MIA mice, n = 24). Student’s unpaired *t* test, p = 0.022. (a, b, d, e): data represented as mean +/- SEM. (c): data represented as box-whisker plot.

Three-chamber testing was used to assess social behavior [39]. During phase I of testing (sociability), mice are presented with the options of interacting with a stranger con-specific mouse or an object. Since typical-behaving mice are social the majority of time is spent exploring the other mouse, while atypical socially-impaired mice show either equal preference for both mouse and object or may show stronger preference for the object. The results of three-chamber testing revealed significant social behavioral deficits in the MIA mice compared to controls. Control mice had an average sociability index score of 29.5 ± 1.3, while MIA mice had a significantly lower sociability index score of 6.7 ± 1.3; p < 0.0001 (Fig 1A)—a score approaching social indifference.

During phase II of three-chamber testing (social novelty), the object was replaced by a stranger mouse. Typical-behaving mice show more interest in the stranger mouse than the familiar mouse, while atypical socially-impaired mice may have social memory impairments and spend equal time with both the familiar and stranger mice [40]. Phase II testing revealed that MIA mice exhibit social novelty deficits in relation to control mice, with index scores of 8.6 ± 2.2 and 17.5 ± 2.1, respectively; p = 0.0077 (Fig 1B). Of note, both normal mice and MIA mice showed increased variability in their social novelty behavioral metrics.

In order to assess communication, USVs were recorded during male-female interaction pairings. MIA male mice were placed into a fresh cage with an unfamiliar control C57BL/6J female mouse in estrous. USVs were recorded over a five-minute period and the number of USVs produced was quantified following completion of the session. The results showed that MIA mice had a significantly decreased incidence of USV emissions compared to controls, with control C57BL/6J mice emitted USVs at 523 ± 117 compared to MIA mice at 101 ± 36, p < 0.001 (Fig 1C). Repetitive behavior was assessed by two behavioral tests. Repetitive behavior as assessed through percentage of marbles buried over a thirty-minute period was comparable between the control and MIA mice, with an average of 50.2 ± 5.8 percent buried by control mice and 48.9 ± 6.3 percent buried by MIA mice; p = 0.88 (Fig 1D). Repetitive behavior was also assessed by self-grooming testing, which was performed by habituating the test mouse to a beaker followed by observation of the time spent grooming in the beaker, with increased grooming time indicative of more repetitive behavior. MIA mice exhibited significantly more grooming time compared to control mice at 153.3 ± 14.3 seconds compared to 105 ± 11.2 seconds, respectively; p = 0.022 (Fig 1E). In summary, MIA mice demonstrated core behavioral features associated with ASD including impaired social interaction, decreased communication, and more repetitive behavior.

### Melanotan-II treatment improved sociability deficits in MIA mice

MIA mice were treated with continuous 7-day intraventricular infusion of either MT-II or normal saline. The intraventricular route was used to ensure effective delivery of MT-II with maintenance of continuous levels throughout the central nervous system. Social behavior was evaluated using three-chamber testing.

**Fig 2:**
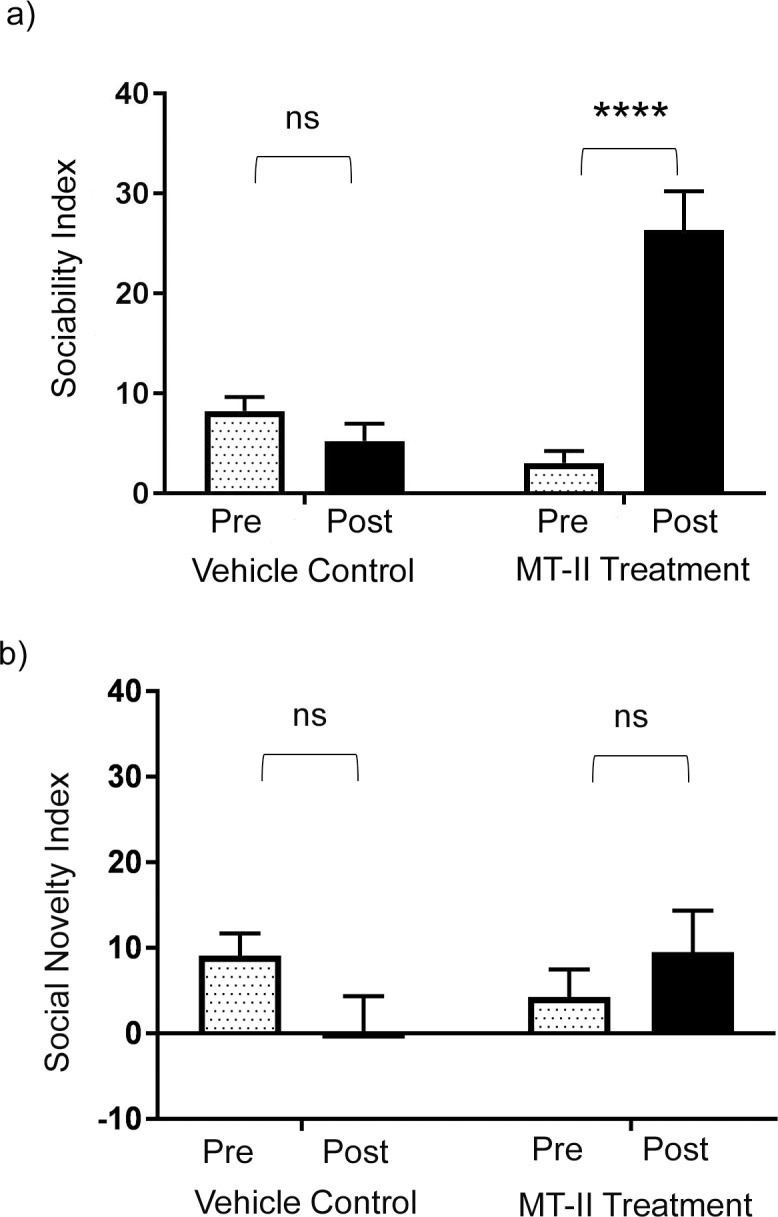
Melanotan-II improved sociability index scores but did not affect social novelty scores. (a) Three-chamber test assessment of sociability index, with results shown for MIA mice pre- and post-vehicle control or pre- and post- MT-II treatment (vehicle, n = 7; MT-II treatment, n = 10). One-way ANOVA comparison between MIA mice treated by vehicle versus MT-II showed a significant treatment interaction between groups: F(_3,30_) = 19.33; p < 0.0001. Sidak’s multiple comparison test showed no significant increase in sociability index following vehicle administration in MIA mice; p = 0.70. Sidak’s multiple comparison test showed a significant increase in sociability index following MT-II administration in MIA mice; p < 0.0001. (b) Three-chamber test assessment of social novelty index, with results shown for MIA mice pre- and post-vehicle control or MT-II treatment (vehicle, n = 7; MT-II treatment, n = 8). One-way ANOVA comparison between MIA mice treated by vehicle versus MT-II did not show a significant treatment interaction between groups: F(_3,26_) = 1.28; p = 0.30. Sidak’s multiple comparison test did not show any significant changes in social novelty in the vehicle treated MIA mice; p = 0.23 or the MT-II treated MIA mice; p = 0.55. (a, b): data represented as mean +/- SEM.

Pre-MT-II treatment, the MIA mice had low baseline sociability index scores of 3.1 ± 1.2, while post-MT-II treatment the sociability index increased significantly to 26.3 ± 3.9 (p < 0.0001) (Fig 2A), achieving a sociability index comparable to levels found in control C57BL/6J mice reported above (29.5 ± 1.3; p = 0.33) (Fig 1A). Vehicle-control MIA mice that received intraventricular normal saline infusions did not have a significant change in sociability index scores, with pre-vehicle scores of 8.2 ± 1.4 comparable to post-vehicle score of 5.2 ± 1.7, p = 0.70 (Fig 2A). One-way ANOVA analysis comparing drug treatment effects between the two MIA mouse groups showed a significant treatment interaction F(_3, 30_) = 19.3; p < 0.0001. The MIA mice did not show a significant increase in their social novelty scores following MT-II administration, with pre-MT-II treatment scores of 4.23 ± 3.2 and post-MT-II treatment scores of 10.7 ± 5.4, p = 0.55 (Fig 2B). Vehicle-control MIA mice exhibited no significant change in social novelty, with pre-vehicle scores of 9.1 ± 2.6 and post-vehicle scores of 0.13 ± 4.2, p = 0.23 (Fig 2B). One-way ANOVA analysis comparing drug treatment effects between the two MIA mouse groups did not show a significant treatment interaction F(_3, 26_) = 1.28; p = 0.30.

### Melanotan-II treatment did not affect baseline social behavior in normal C57BL/6J mice

To evaluate the behavioral effects of MT-II on the normal C57BL/6J mouse strain, MT-II was administered by continuous intraventricular infusion over a 14-day course. Vehicle-control mice were infused with normal saline for comparison. By extending the length of infusion, we were able to evaluate for any potential MT-II associated side effects or changes affecting weight or behavior in normal C57BL/6J mice. Analysis included three chamber testing as discussed in this section, marble burying, exploratory behavior assessed by the open field anxiety test, and weight changes as discussed in the following section. One week before implantation of the intraventricular pumps, mice underwent baseline testing. Subsequent testing following infusion of MT-II or normal saline was done on the indicated days post-infusion.

**Fig 3:**
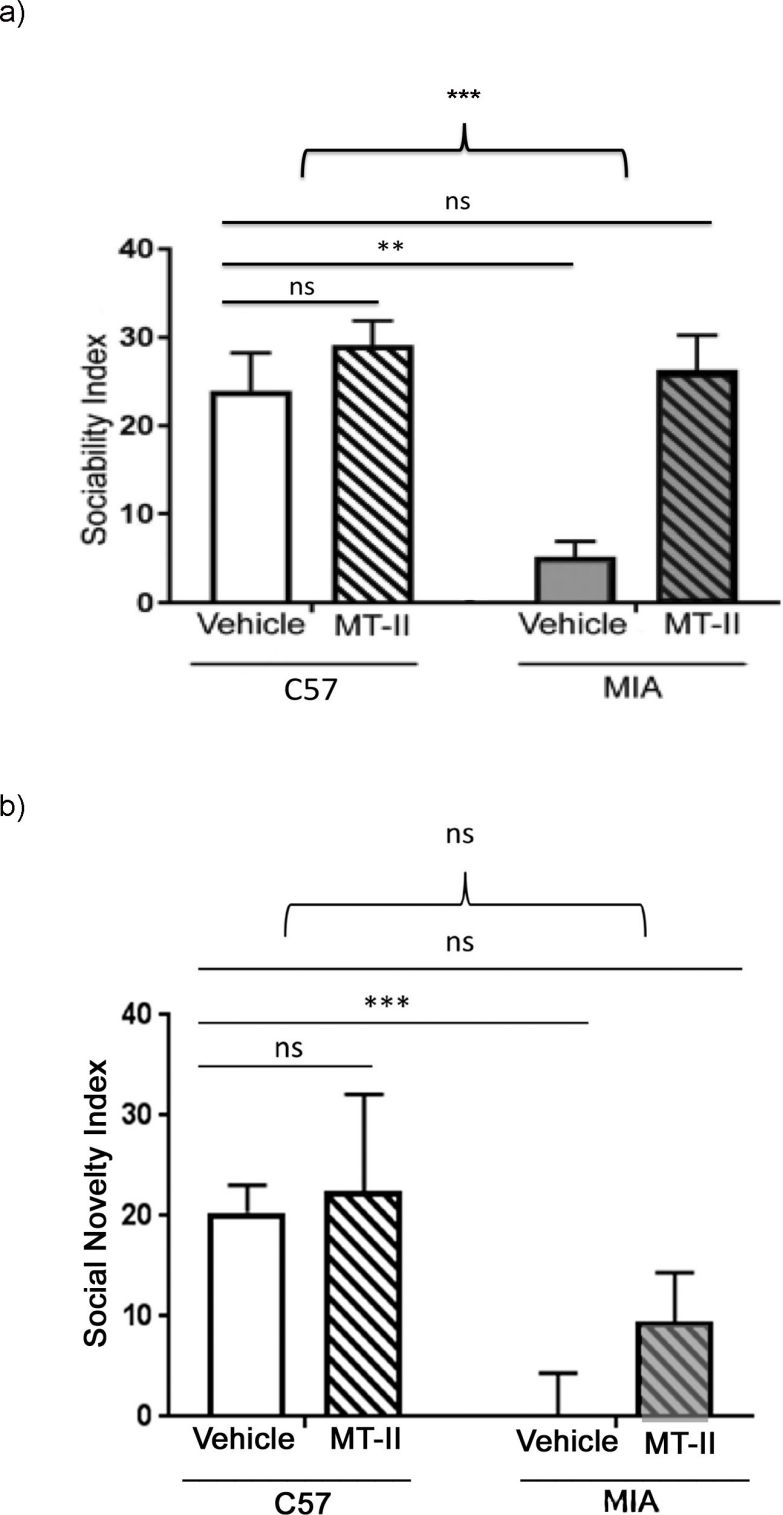
Melanotan-II did not significantly alter social behaviors in normal C57BL/6J male mice. (a) Three-chamber test assessment of sociability index, with results shown for normal C57BL/6J mice following vehicle control (n = 7) or MT-II treatment (n = 9), and MIA mice following vehicle control (n = 7) or MT-II treatment (n = 10). Student’s unpaired *t* test analysis was used to compare vehicle-control normal C57BL/6J mice to MT-II-treated normal C57BL/6J mice (p = 0.77), vehicle-control MIA mice (p = 0.0017), and MT-II-treated MIA mice (p = 0.68). The above bracket represents a two-way ANOVA analysis of treatment interaction between the normal C57BL/6J mice and MIA mice; interaction F (_1,29_) = 14.34, p = 0.0007. (b) Three-chamber test assessment of social novelty, with results shown for normal C57BL/6J mice following vehicle control (n = 7) or MT-II treatment (n = 6), and MIA mice following vehicle control (n = 7) or MT-II treatment (n = 8). Student’s unpaired *t* test analysis was used to compare vehicle-control normal C57BL/6J mice to MT-II-treated normal C57BL/6J mice (p = 0.88), vehicle-control MIA mice (p = 0.0002), and MT-II-treated MIA mice (p = 0.16). The above bracket represents a two-way ANOVA analysis of treatment interaction between the normal C57BL/6J mice and MIA mice; interaction F (_1,24_) = 0.43, p = 0.52. (a, b): data represented as mean +/- SEM.

Given the above noted effect of MT-II on social behavior in MIA mice, we were interested in assessing the effects of MT-II on social behavior in normal C57BL/6J mice. Three-chamber testing was performed to evaluate the effect of MT-II on social behavior of normal C57BL/6J mice. Following 7 days of MT-II treatment, there was no significant change in sociability index, with post MT-II treatment sociability index scores of 29.1 ± 2.8 and post-vehicle treatment scores of 23.9 ± 4.3; p = 0.77 (Fig 3A). Comparison of the vehicle-treated normal C57BL/6J mice to vehicle-treated MIA mice showed significantly lower sociability index scores in the MIA mice, p = 0.0017. In contrast, comparison between the vehicle-treated normal C57BL/6J mice and the MT-II-treated MIA mice did not show a significant difference in sociability index scores, p = 0.69. This finding is consistent with the MT-II treatment rescuing the sociability behavior of the MIA mice. A two-way ANOVA comparison between the normal C57BL/6J mice and the MIA mice detected a significant MT-II treatment interaction F(_1, 29_) = 14.3; p = 0.0007 (Fig 3A).

MT-II treatment did not significantly affect social novelty scores in the normal C57BL/6J mice with post-MT-II treatment social novelty index at 22.3 ± 9.7 and post-vehicle treatment scores at 20.3 ± 2.7; p = 0.88 (Fig 3B). Comparison of the vehicle-treated normal C57BL/6J mice to vehicle-treated MIA mice showed significantly lower social novelty index scores in the MIA mice, p = 0.0002. Also, comparison of the vehicle-treated normal C57BL/6J mice and the MT-II-treated MIA mice did not show a significant difference in social novelty index scores, p = 0.16. A two-way ANOVA comparison evaluating for social novelty treatment interaction between normal C57BL/6J mice and the MIA mice did not detect a significant interaction F(_1, 24_) = 0.43; p = 0.52 (Fig 3B). The lack of treatment effect is likely due to increased variability of social novelty index scores. In summary, this data showed no significant difference in the sociability and social novelty behaviors of normal C57BL/6J mice following MT-II treatment. Furthermore, this data shows that administration of MT-II to the MIA treated mice rescued their sociability deficits. Comparable social novelty scores were seen between vehicle-treated normal C57BL/6J mice and MT-II treated MIA mice, though a significant treatment interaction was not observed between the two groups.

### Melanotan-II treatment resulted in weight loss but did not affect repetitive or anxiety-like behavior of normal C57BL/6J mice

MC4R stimulation has been linked to increased stress behaviors based on its effects on the hypothalamic-pituitary-adrenal axis [41]. For instance, MC4R intracerebroventricular administration has been shown to induce the gene transcription of cortisol releasing hormone with effects blocked by administration of an MC4R antagonist [42]. Also, previous reports have shown that acute treatment with MC4R agonists results in increased repetitive behavior and increased anxiety-like behavior in both the presence and absence of psychological stress [43, 44, 45]. Additionally, MC4R activation is well characterized to suppress appetite [46]. We were interested in evaluating for possible adverse effects of MT-II including anxiety-like and repetitive behavior, in particular over a subacute period on the order days as opposed to acute treatment on the order of hours as in previous reports [45]. While previous investigators studied MC4R agonist effects on anxiety-like behavior following induced stress of restraints or forced swimming, we were interested in anxiety-like behavior without incurring psychological stress.

**Fig 4:**
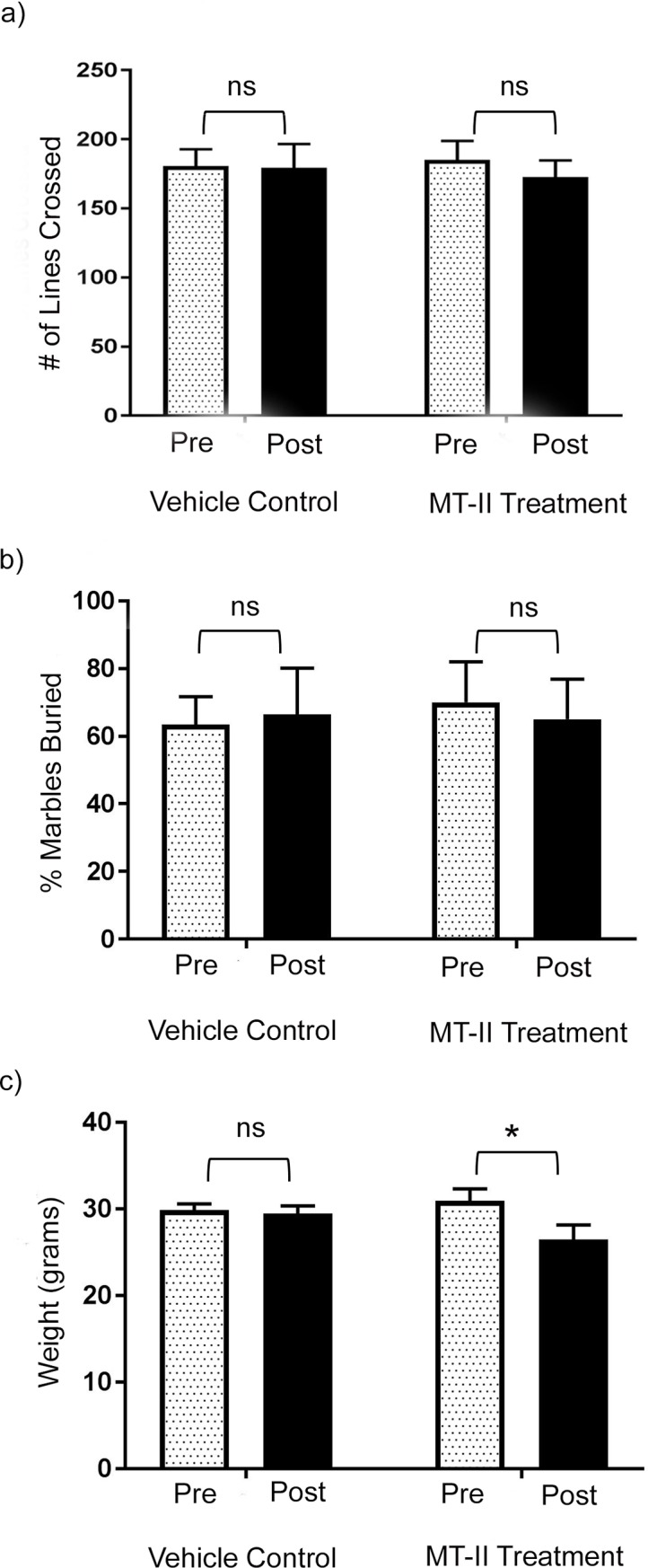
Melanotan-II did not significantly impact exploratory behavior or marble burying behavior, but caused significant weight loss in C57BL/6J mice. (a) Open field anxiety test quantified as number of gridlines crossed, with results shown for normal C57BL/6J mice pre- and post-vehicle control or MT-II treatment for 9 days (vehicle, n = 7; MT-II treatment, n = 9). ANOVA comparison between normal C57BL/6J mice treated with vehicle versus MT-II did not show a significant treatment interaction in regards to number of gridlines crossed; F(_3, 28_) = 0.89. Sidak’s multiple comparison tests did not show a significant change in open field anxiety test performance in the vehicle treated mice (p = 0.97) or MT-II treated mice (p = 0.73). (b) Marble burying testing quantified as the percentage of marbles buried, with results shown for normal C57BL/6J mice pre- and post-vehicle control or MT-II treatment for 14 days (vehicle, n = 7; MT-II treatment, n = 7). ANOVA comparison between normal C57BL/6J mice treated with vehicle versus MT-II did not show a significant treatment interaction with regards to marble burying; F(_3, 24_) = 0.067; p = 0.97. Sidak’s multiple comparison tests did not show a significant change in marble burying in the vehicle treated mice (p = 0.97) or MT-II treated mice (p = 0.94). (c) Weight in grams shown for normal C57BL/6J mice pre- and post-vehicle control or MT-II treatment for 14 days (vehicle, n = 7; MT-II treatment, n = 7). ANOVA comparison between normal C57BL/6J mice treated with vehicle versus MT-II did not show a significant treatment interaction with regards to weight loss; F(_3, 24_) = 2.35; p = 0.098. Sidak’s multiple comparison test showed no significant change in the vehicle treated mice; p = 0.97. Sidak’s multiple comparison test showed significant weight loss in the MT-II treated mice; p = 0.037. (a-c): data represented as mean +/- SEM.

Open field anxiety testing was used to assess exploratory behavior, a measure of anxiety-like behavior, with scoring based on the number of grid line crossings. Baseline testing was done one week prior to intraventricular infusion of MT-II or vehicle control, and repeat testing was performed on day nine of infusion. The results of open field anxiety testing showed no difference in exploratory behavior in mice pre-MT-II treatment (185 ± 13 crossings) compared to post-MT-II treatment (173 ± 12 crossings); p = 0.73 (Fig 4A). There was also no significant difference in mice pre-vehicle control exploration (180 ± 12 crossings) and in post-vehicle controls (179 ± 17 crossings); p = 0.97 (Fig 4A). The ANOVA did not reveal evidence of a significant treatment interaction between the two groups of mice, F(_3, 28_) = 0.27; p = 0.89. Repetitive behavior was analyzed by marble burying testing on post-infusion day 14. Marble burying testing showed no significant difference in the percentage of marbles buried at baseline (70 ± 12) and post-MT-II (65 ± 11); p = 0.94 (Fig 4B). The vehicle-treated mice also did not show a significant difference in the percentage of marbles buried with a baseline at 63 ± 8 percent and post-treatment at 66 ± 13 percent; p = 0.97 (Fig 4B). The ANOVA did not reveal evidence of a significant treatment interaction between the two groups of mice, F(_3, 24_) = 0.067; p = 0.98. Consistent with previous literature findings of weight loss in rodents following MC4R stimulation [47], mice treated with MT-II demonstrated significant weight loss over the 14-day treatment course. Baseline weight decreased from 30.9 ± 1.4 grams to 26.5 ± 1.7 grams following MT-II treatment; p = 0.037 (Fig 4C). Vehicle control C57BL/6J mice maintained comparable weights at the end of the 14-day course, with baseline weight of 29.8 ± 0.7 grams compared to post-vehicle control weight of 29.4 ± 0.8 grams; p = 0.97 (Fig 4C). There was no significant ANOVA treatment interaction identified between both groups of mice, F (_3, 24_) = 2.346; p = 0.098.

### Male MIA mice exhibited increased oxytocin receptor expression in the anterior cingulate cortex compared to normal C57BL/6J mice

Currently, limited data in the literature suggests a possible role for IL-6 in the regulation of oxytocin receptor expression. Schmid et al. [48] demonstrated that the oxytocin receptor gene promoter is negatively regulated by IL-1β and IL-6 in functional transfection studies involving HeLA cells. Furthermore, exposure of myometrial cells to IL-1β or IL-6 resulted in a significant reduction of oxytocin receptor expression. Rauk et al. [49] observed an opposite effect with up-regulation of oxytocin receptor expression in primary culture of human myometrium exposed to IL-6. Currently, it is unclear if there is a direct effect of IL-6 on the development of the oxytocin system in the brain. Of interest, administration of IL-6 occurs at E12.5–16.5 corresponding approximately with the onset of oxytocin receptor system development in the male mouse brain at E16.5. Since the male MIA mice were sensitive to endogenous oxytocin production, we assessed for any potential differences in oxytocin receptor expression in the adult MIA male mice and compared the results to the normal C57BL6/J mice. Oxytocin receptor density was evaluated in the prelimbic cortex, anterior olfactory nucleus, nucleus accumbens, lateral septal nucleus, bed nucleus of the stria terminalis, ventral reuniens, paraventricular, and ventromedial hypothalamic nucleus.

**Fig 5:**
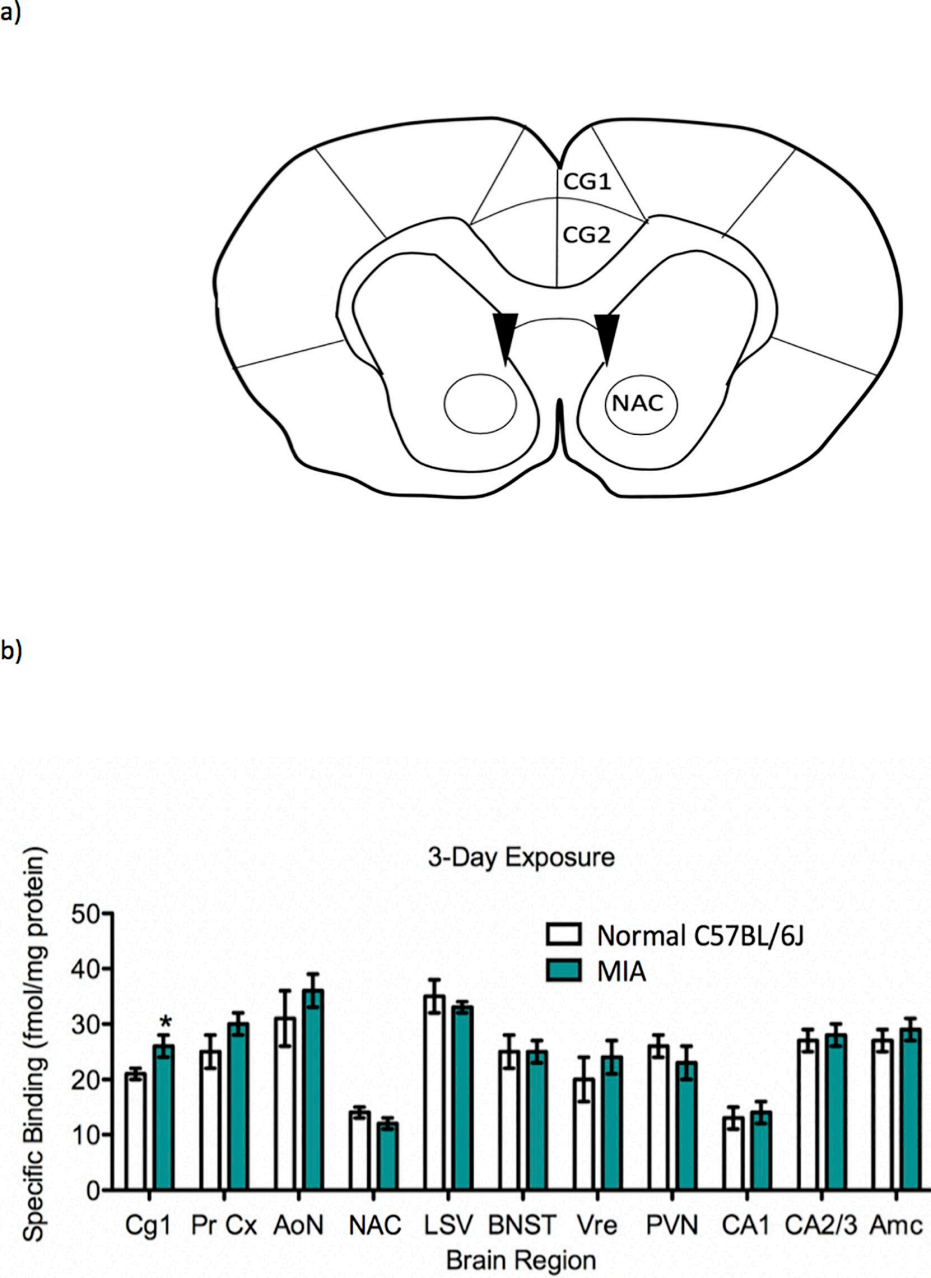
Adult male MIA mice demonstrated an increase in oxytocin receptor expression in the anterior cingulate cortex. (a) A coronal section demonstrating the cingulate cortex region 1 (CG1), cingulate cortex region 2 (CG2), and nucleus accumbens (NAC) in the mouse brain. (b) Oxytocin receptor autoradiography demonstrating the comparison of oxytocin receptor binding (fmol/mg protein) between the normal C57BL/6J male mouse brains and the MIA male mouse brains. The following high oxytocin receptor brain regions were evaluated: CG1 (cingulate cortex region 1), perirhinal cortex (PRCx), anterior olfactory nucleus (AoN), nucleus accumbens (NAC), lateral septal nucleus (LSV), bed nucleus of the stria terminalis (BNST), ventral reuniens nucleus (Vre), paraventricular nucleus (PVN), hippocampal CA1 and CA2/3 regions, and the central amygdaloid nucleus (Amc). Oxytocin receptor binding site density was significantly elevated (df = 8, t = -2.63, p < 0.05) in the cingulate cortex region 1 (including the anterior cingulate cortex; ACC) of MIA mice (26 ± 2 mol/mg protein; n = 5) relative to normal C57BL/6J mice (21 ± 1 fmol/mg protein; n = 5). Multivariate Analysis of Variance with Newman Keuls post-hoc was performed to compare oxytocin receptor expression in the brain regions of interest between untreated MIA mice and normal C57BL6/J mice groups.

Oxytocin receptor binding site density was significantly elevated (df = 8, t = -2.63, p < 0.05) in the cingulate cortex region 1 (including the anterior cingulate cortex; ACC Fig 5A) of MIA mice (26 ± 2 fmol/mg protein; n = 5) relative to normal C57BL/6J mice (21 ± 1 fmol/mg protein; n = 5) as seen in Fig 5B. There were no other significant differences in oxytocin receptor density between groups in any other high expression oxytocin receptor brain regions, which included prelimbic cortex, anterior olfactory nucleus, nucleus accumbens, lateral septal nucleus, bed nucleus of stria terminalis, ventral reuniens, paraventricular and ventromedial hypothalamic nuclei, hippocampal CA1 and CA2/3 regions and the central amygdaloid nucleus.

## Discussion

Our studies show that 1) administration of MT-II to MIA male mice significantly improved sociability indices but did not affect social novelty scores following intra-ventricular administration; 2) administration of MT-II to normal C57BL/6J mice did not affect sociability and social novelty scores; 3) subacute administration of the MC4R agonist did not lead to increased repetitive or anxiety-like behavior in the normal C57BL/6J mice, though did result in weight loss; 4) oxytocin receptor expression was upregulated in the anterior cingulate cortex of the adult male MIA mice compared to typical-behaving C57BL/6J male mice.

Several behavioral studies have documented enhancement of social cognition with administration of MT-II. Experiments performed on female prairie voles demonstrated that social isolation during early life impaired partner pair preferences as adults [50]. However, if the pups were socially isolated but received daily peripheral MT-II injections during the first seven days of the fourteen-day isolation period, the female prairie voles maintained normal partner preferences as adults. Another study demonstrated that intra-peritoneal injection of MT-II in adult female prairie voles increased partner preference formation through an oxytocin-dependent mechanism of action [20]. Given the promising findings in the above studies, we were interested in evaluating the effect of MT-II on social cognition in autism-like and normal male C57BL/6J mice. This study is the first one to our knowledge, which extensively evaluates the effects of MT-II on social behaviors of an environmental autism-like mouse model and normal C57BL/6J mice.

Administration of MT-II to MIA male mice resulted in significantly improved sociability indices. The sociability behaviors of the MIA mice normalized to levels found in normal C57BL/6J. Our findings are suggestive of an oxytocin/oxytocin receptor signaling abnormality in the MIA mice. Performance of oxytocin receptor autoradiography of the brain identified an increase in oxytocin receptor density in the anterior cingulate cortex (ACC) of the male MIA mice compared to the typical-behaving C57BL/6J mice. The ACC is involved in complex cognitive functions, including empathy, emotion, and decision making. The findings of increased oxytocin receptor density in the ACC within the MIA mice is of interest, as recent studies have identified oxytocin signaling as mediating rodent empathy and anxiety behaviors through the ACC. According to Burkett et al. [51], consolation behaviors defined as “an increase in affiliative contact in response to and directed toward a distressed individual” were shown to increase in prairie voles through the stimulation of oxytocin receptors within the ACC. Furthermore, a recent paper by Li et al. [52] identified a cortical circuit for sexually dimorphic oxytocin-dependent anxiety behaviors. Specifically, they found that activation of oxytocin receptor inter-neurons within the pre-frontal cortex of the ACC by oxytocin had an anxiolytic effect on male but not female C57BL/6J mice. Oxytocin stimulation of the oxytocin interneurons resulted in production of GABA and cortisol release hormone binding protein (CRHBP), which inhibited the stress/anxiety behavior mediated by cortisol releasing hormone within layer 2/3 pyramidal neurons associated with cortisol releasing hormone release.

The significance of increased oxytocin receptor expression in the ACC of MIA mice is suggestive of a possible circuit involved in mediating social behavior via an oxytocin-dependent mechanism. Since the administration of the rIL-6 coincides with the embryonic development of the oxytocin signaling system, it is unclear if rIL-6 or its downstream mediators may have a long-term impact on oxytocin receptor expression in the adult MIA male mouse. Perhaps the up-regulation of oxytocin receptor expression in the ACC allows for MT-II to mediate socio-emotional processing and results in the increased sociability of the MIA mice secondary to downstream endogenous oxytocin release. Though our current data demonstrates an association of socially-impaired MIA mice and increased oxytocin receptor binding density in the ACC, further investigation is needed to establish whether the ACC is involved in regulating differences in social behavior observed in MIA mice and control C57BL/6J mice. To prove the exact underlying baseline neuro-circuitry within the ACC of MIA mice versus C57BL/6J mice would require electrophysiological records from the ACC oxytocin neurons at baseline in the MIA and normal C57BL/6J mice along with any potential changes that may occur with MT-II administration. We find the above result intriguing given the recent rodent behavioral research implicating the pre-frontal cortex and the ACC as a key socio-emotional processing center requiring oxytocin for social salience.

We believe that the social deficits in autistic adult MIA mice are likely due to structural changes with aberrant neurotransmitter activity. Given that improvement in social deficits is observed following relatively short-term MT-II treatment, this is likely mediated by neuromodulatory signaling downstream of MC4R including increased oxytocin activity that overrides existing asocial structural abnormalities. Canetta et al. [53] found that adult MIA mice had decreased GABAergic transmission from parvalbumin interneurons synapsing on pyramidal layer 2/3 of the medial prefrontal cortex. Abnormalities in the spiking of GABAergic interneurons within the prefrontal cortex has been implicated in the pathophysiology of multiple psychiatric disorders including schizophrenia. The researchers demonstrated that inhibition of the paravalbumin GABAergic interneurons resulted in increased anxiety behavior in the MIA mice. Of interest, oxytocin has been shown to have anxiolytic effects in the pre-frontal cortex through binding of oxytocin receptors [54]. Meanwhile, Li et al. [55] showed that MIA mice had increased synaptic strength in glutamatergic projections from the medial prefrontal cortex to the basolateral nucleus of the amygdala (BLA). Hyper-excitability of the BLA is associated with behavioral disorders and involves excessive fear and anxiety [56]. Campbell-Smith et al. [57] showed that oxytocin administration into the BLA impaired acquisition of context-conditioned fear, demonstrating a neuro-modulatory role for the neuropeptide. Given the above findings, perhaps the downstream effects of endogenous oxytocin stimulation results in alleviation of fear-based responses to social stimuli in the MIA mice. These findings together with our current study suggest a role for MC4R in neuromodulation of MIA mice to improve social behavior through oxytocin, though other downstream signaling pathways may likely be involved and will require further investigation.

Future studies with long-term treatment would be helpful to determine whether chronic MC4R agonism may result in the formation of new pro-social structural changes or even lead to reversal of existing structural abnormalities. MC4R agonism has been shown to induce synaptic changes by increasing AMPA receptors, alterations in dendritic spine densities and increasing sensitivity to glutamate. For instance, MC4R agonism in the nucleus accumbens altered dendritic morphology and increased behavioral sensitization to drugs of abuse. Furthermore, MC4R agonism has also been implicated in learning and memory through LTP enhancement via cAMP-PKA cascades within the hippocampus [58].

Overall, the MIA mice had significant improvement of sociability while receiving the MT-II infusion and this is the first study to our knowledge to explore chronic administration of melanocortin 4 receptor agonist on social behaviors. Future studies will focus on exploration of alternate modalities for drug delivery as well as duration of behavioral effect following cessation of MT-II infusion. Of note, prior to attempting intra-ventricular infusion of MT-II, we tried multiple different regimens of MT-II intra-peritoneal injections in the MIA mice. Preliminary findings using different scheduling regiments such as once daily or twice daily intra-peritoneal injections of MT-II over seven days did not yield any significant difference in social behavior. Furthermore, testing for any potential acute behavioral effects within 30 minutes of the intra-peritoneal injection also did not yield any significant findings. This is contrast to previous findings of acute MC4R peripheral administration leading to changes in social behavior as previously described by Penagarikano et al. [12] in the Cntnap 2 mouse model of autism. A potential explanation for the observed difference could be secondary to the age of the mice at which the melanocortin agonist was administered. The Cntnap2 KO male mice were juveniles at 4–6 weeks of age unlike the MIA male mice who were aged 4–6 months. A previous study from Douglas et al. [59] evaluated social rewarding properties of social interactions and included comparisons between juvenile and adult rats. They found that isolated juvenile and adult rats developed social conditioned place preference with the strongest preference found in juvenile males. Social conditioned place preference was not evident in group-housed adults whereas group-housed adolescents developed a preference for the compartment previously paired with similarly housed partners. The findings are suggestive of social interactions being more rewarding for juveniles. A recent publication by Smith et al. [60] evaluated for potential differences in oxytocin receptor expression and distribution in the brain as a potential explanation for differences between juvenile versus adult rat behavior governing social interactions. They noted some of the marked differences were increased oxytocin receptor binding density in the nucleus accumbens and dorsal caudate putamen in the juvenile rats compared to adult rats in both males and females. These two brain regions have been implicated in mediating socially rewarding behaviors. As such, perhaps the ability of the juvenile Cntnap2 KO mice to respond acutely to the MC4R agonist may be secondary to a critical time point in development with increased responsiveness to oxytocin stimulation and reward behaviors based on oxytocin receptor distribution. However, the adult mouse may have less oxytocin receptor distribution in regions governing socially rewarding behaviors and continuous stimulation of oxytocin release is necessary to obtain an effect. We also theorize that due to the relatively short-half-life of MT-II (approximately 2 hours), once or twice daily dosing may not be sufficient to meet a threshold at which effective neuro-modulation may occur in adult C57BL/6J mice. However, we did note based on preliminary testing that the effect of the MT-II intraventricular infusion normalized the sociability index of mice as early as 48 hours into treatment.

MT-II treatment of normal C57BL/6J mice did not affect sociability and social novelty index scores as compared to baseline values. This finding is suggestive of an adequately saturated oxytocin signaling system given increased stimulation of the oxytocin system did not cause significant changes. Similar findings were observed by Penagarikano et al. [12], as wild-type mice continued to show similar social behaviors in the setting of oxytocin treatment.

We evaluated for any potential behavioral side effects such as anxiety-like behavior or repetitive behaviors with MT-II in the normal C57BL/6J mice given the known role of acute MC4R stimulation in increasing stress behaviors in rodents. For instance Liu et al. [44] demonstrated that acute administration of a MC4R agonist into the medial amygdala in the setting of restraint stress resulted in increased anxiety behavior in rats accompanied by increased c-Fos mRNA expression in medial amygdala MC4R neurons. We used the open-field anxiety test as a broad screen to evaluate the effects of subacute MT-II infusion on mouse anxiety-like behavior. Our findings showed that there was no significant difference in exploratory behavior as assessed by the open-field anxiety test following subacute infusion of MT-II or vehicle control. These findings are consistent with a previous study [45], which showed acute intra-peritoneal MT-II administration in rats that experienced a stress stimulus resulted in no significant difference in locomotion, exploratory behavior, or freezing behavior in the open-field anxiety test. We also did not see a significant change in percentage of marbles buried in the MT-II treated mice or the control group.

We evaluated for weight loss as a known non-behavioral side effect of MT-II, since stimulation of the MC4R system has been linked to suppression of appetite. Both rodent and obese rhesus macaques demonstrated weight loss and increased energy expenditure with MC4R stimulation [61]. Consistent with other studies, we also found significant weight loss in the MT-II treated mice while the vehicle controls maintained baseline weights 14 days into the infusion. Measurement of weight loss served as an effective secondary measure for evaluating adequate drug delivery in addition to cannula placement confirmation as assessed through brain paraffin sections.

Oxytocin blood concentration and oxytocin receptor genotypes have been shown to correlate generally with social impairment [9]. Several studies, including the current, suggest that stimulation of the oxytocin system may improve social deficits in certain models of ASD. The underlying pathology for ASD is likely heterogeneous, and individuals would be expected to have varying responses to therapies. MC4R agonists are promising candidates for further clinical investigation as a means for stimulating the oxytocin system. Given MC4R targets several organ systems with various physiologic effects, including possible therapeutic roles in promoting weight loss and treating impotence, there are currently several MC4R agonists entering human clinical testing. If these clinical trials show limited toxicity, the potential use of MC4R for ASD human research clinical trials may be a prominent step forward as a potential novel neurotherapeutic agent for the treatment of this complex disorder.

This study is the first to our knowledge to show the promising effects of MT-II treatment in an environmental, non-genetic, mouse model of autism resulting in rescue of social deficits. Notably these effects of MT-II were observed upon treatment of adult mice, suggesting that the potential benefits of treatment may not be limited to a therapeutic window during early development. The current study expands the application of pharmacologic MC4R stimulation by demonstrating that the MIA mouse model of autism is responsive to MT-II treatment, suggesting a promising approach for further clinical investigation regarding individuals with *in utero* exposure to infection.
